# The paxillin-plectin-EPLIN complex promotes apical elimination of RasV12-transformed cells by modulating HDAC6-regulated tubulin acetylation

**DOI:** 10.1038/s41598-018-20146-1

**Published:** 2018-02-01

**Authors:** Nobuhiro Kasai, Ailijiang Kadeer, Mihoko Kajita, Sayaka Saitoh, Susumu Ishikawa, Takeshi Maruyama, Yasuyuki Fujita

**Affiliations:** 1 Division of Molecular Oncology, Institute for Genetic Medicine, Hokkaido University, Sapporo, 060-0815 Japan; 2 Graduate School of Chemical Sciences and Engineering, Hokkaido University, Sapporo, 060-0815 Japan; 3 Present Address: Department of Endocrinology, Fifth Affiliated Hospital of Xinjiang, Medical University, Urumqi, 830011 China

## Abstract

Recent studies have revealed that newly emerging RasV12-transformed cells are often apically extruded from the epithelial layer. During this cancer preventive process, cytoskeletal proteins plectin and Epithelial Protein Lost In Neoplasm (EPLIN) are accumulated in RasV12 cells that are surrounded by normal cells, which positively regulate the apical elimination of transformed cells. However, the downstream regulators of the plectin-EPLIN complex remain to be identified. In this study, we have found that paxillin binds to EPLIN specifically in the mix culture of normal and RasV12-transformed cells. In addition, paxillin is accumulated in RasV12 cells surrounded by normal cells. Paxillin, plectin and EPLIN mutually influence their non-cell-autonomous accumulation, and paxillin plays a crucial role in apical extrusion of RasV12 cells. We also demonstrate that in RasV12 cells surrounded by normal cells, acetylated tubulin is accumulated. Furthermore, acetylation of tubulin is promoted by paxillin that suppresses the activity of histone deacetylase (HDAC) 6. Collectively, these results indicate that in concert with plectin and EPLIN, paxillin positively regulates apical extrusion of RasV12-transformed cells by promoting microtubule acetylation. This study shed light on the unexplored events occurring at the initial stage of carcinogenesis and would potentially lead to a novel type of cancer preventive medicine.

## Introduction

At the initial stage of carcinogenesis, an oncogenic mutation occurs in single cells within the epithelium. Recent studies have revealed that the newly emerging transformed cells and the surrounding normal epithelial cells often compete with each other for survival^1–10^. This phenomenon is called cell competition; the loser cells are eliminated from epithelial tissues, while the winner cells proliferate and fill the vacant spaces. By using Madin-Darby canine kidney (MDCK) epithelial cells stably expressing RasV12 in a tetracycline-inducible manner, we have demonstrated that when Ras-transformed cells appear within the epithelial monolayer, the transformed cells are extruded into the apical lumen of the epithelium in a cell death-independent fashion, a process called apical extrusion^11^. Together with other studies, it has become evident that normal epithelial cells can recognize and actively eliminate the neighbouring transformed cells from epithelial tissues via cell competition. This cancer preventive mechanism is termed Epithelial Defense Against Cancer (EDAC)^12,13^.

In the cell competition between normal and RasV12-transformed epithelial cells, the presence of normal cells profoundly influences various cellular processes and signalling pathways in the neighbouring transformed cells, which positively regulate their apical extrusion. In the previous studies, we have reported that cytoskeletal proteins plectin and Epithelial Protein Lost In Neoplasm (EPLIN) are accumulated in RasV12 cells when they are surrounded by normal cells^14,15^. The plectin-EPLIN complex then induces α-tubulin polymerization, leading to the accumulation of microtubule filaments. This process plays a crucial role in the apical extrusion of RasV12 cells, however the molecular mechanism of how plectin and EPLIN regulate the organization of microtubules remains unknown.

The structure and physical property of microtubule filaments are dynamically regulated by various mechanisms including acetylation of α-tubulin K40^16,17^. In addition, acetylation of tubulin can also influence a variety of cellular processes including vesicle transport, signalling pathways and cell migration^18,19^. Acetylation of tubulin is catalysed by α-tubulin acetyltransferase (αTAT) 1^20,21^, while deacetylation is mediated by histone deacetylase (HDAC) 6^22,23^ and sirtuin (SIRT) 2^24^. The activity of HDAC6 can be regulated by multiple mechanisms such as suppression by paxillin^25^. Paxillin is one of the key adaptor proteins in the integrin-based focal adhesion complex^26^. But, additionally, paxillin localizes in the cytosol and can play other cellular functions^25^.

In this study, we have found that paxillin is a vital regulator of apical extrusion of RasV12-transformed cells by linking the plectin-EPLIN complex and acetylation of microtubules.

## Results

### Paxillin plays a crucial role in apical elimination of RasV12-transformed cells

**Figure 1:**
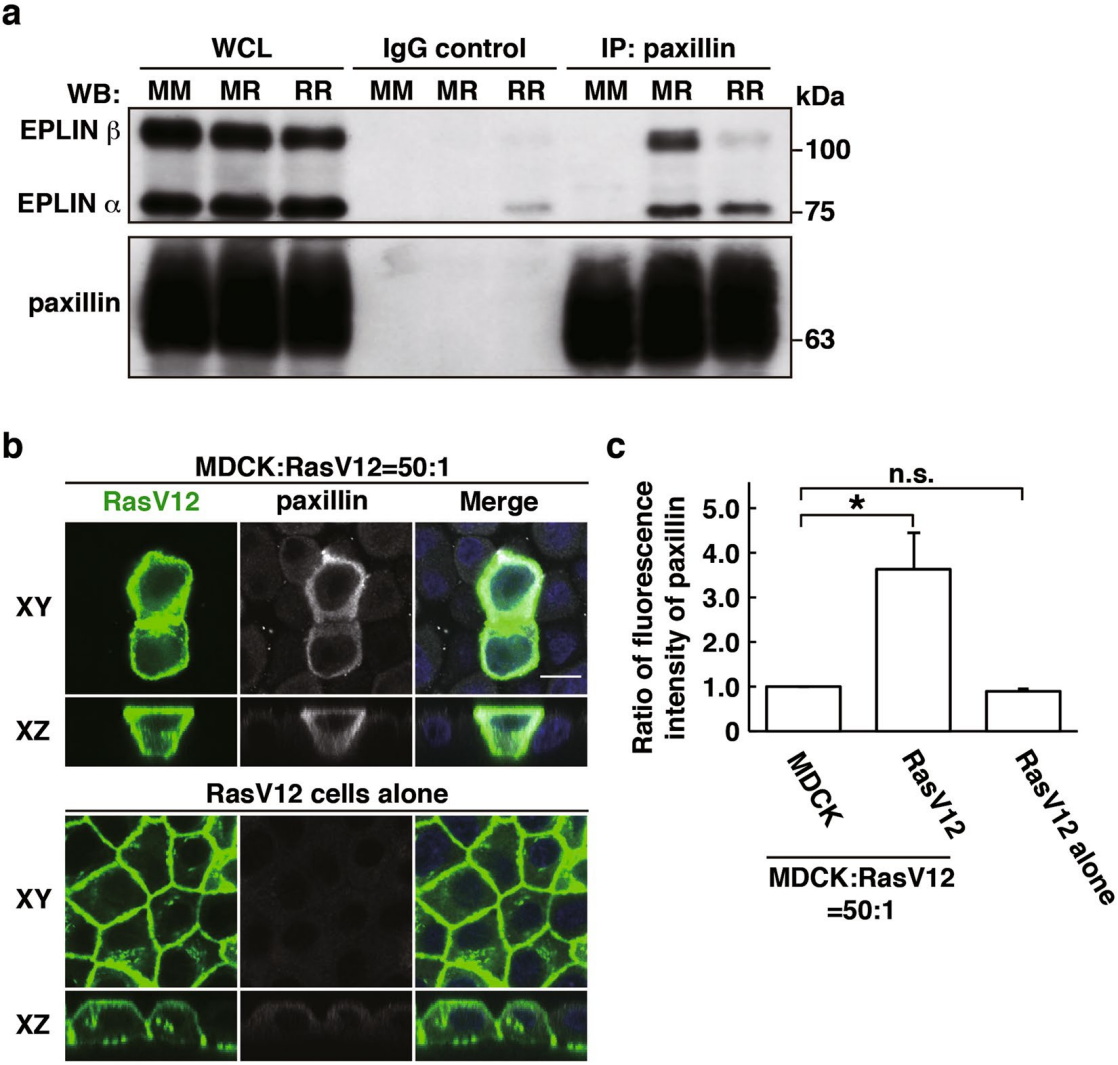
Paxillin is accumulated in RasV12-transformed cells that are surrounded by normal epithelial cells. (**a**) Co-immunoprecipitation of EPLIN with paxillin. MM, normal MDCK cells cultured alone; MR, 1:1 mix culture of normal MDCK and MDCK-pTR GFP-RasV12 cells; RR, MDCK-pTR GFP-RasV12 cells cultured alone. (**b**) Immunofluorescence images of paxillin. MDCK-pTR GFP-RasV12 cells were mixed with normal MDCK cells or cultured alone on collagen gels. Cells were fixed after 16 h incubation with tetracycline and stained with anti-paxillin antibody (grey) and Hoechst (blue). Scale bar, 10 μM. (**c**) Quantification of the fluorescence intensity of paxillin. Data are mean ± SD from three independent experiments. *P < 0.05, n.s., not significant; n ≧ 30 cells for each experimental condition. Values are expressed as a ratio relative to MDCK cells. Note that accumulation of paxillin is also observed in the 1:1 mixed culture by immunofluorescence, but rather moderately. In the 1:1 mixed culture condition, both normal and transformed cells are included, and certain fractions of transformed cells do not directly interact with normal cells. Therefore, non-cell-autonomous changes in transformed cells are diluted by the presence of normal cells and transformed cells that do not interact with normal cells, and thus often difficult to be detected biochemically in the 1:1 mixed culture condition.

**Figure 2:**
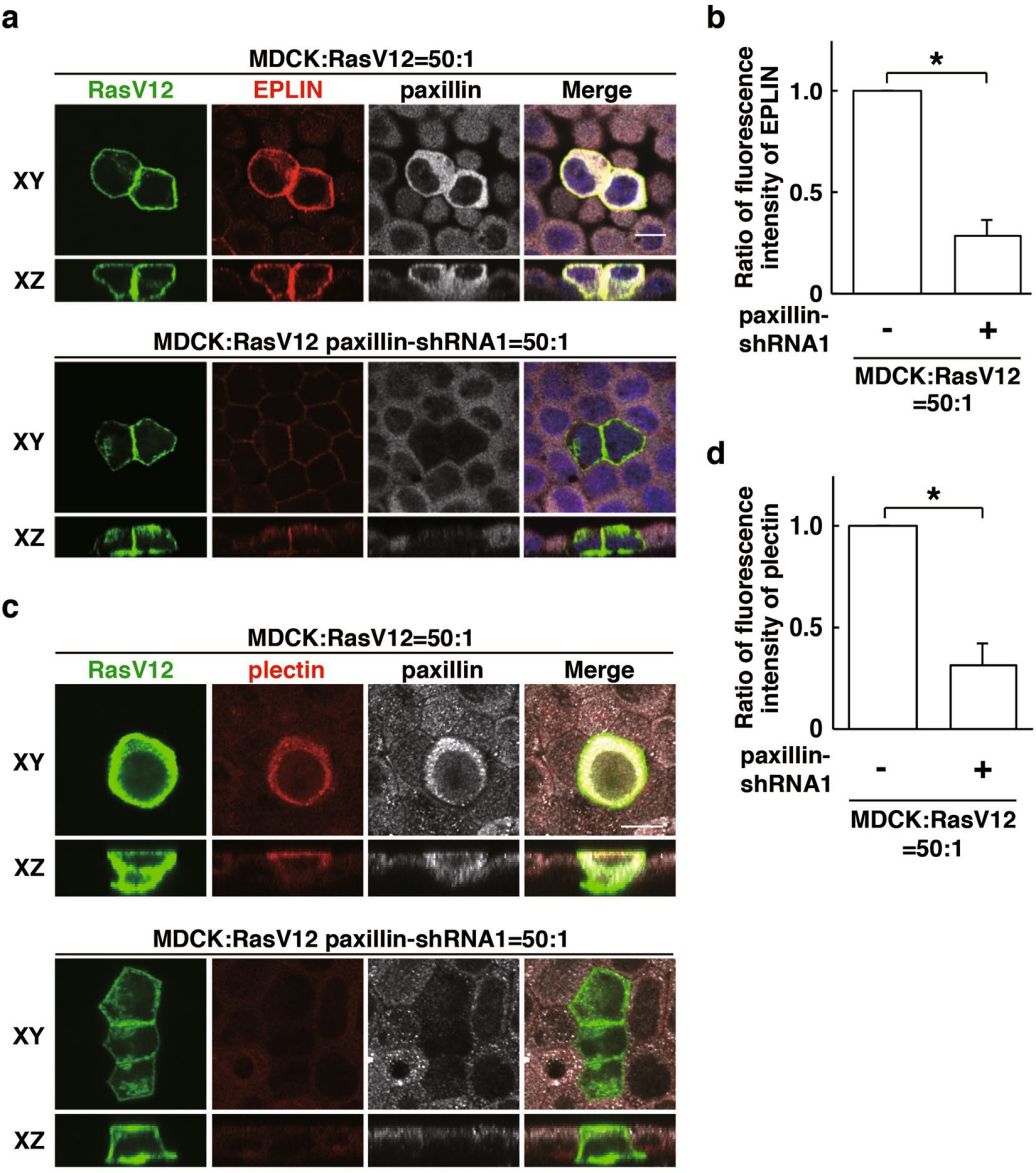
Paxillin regulates accumulation of EPLIN and plectin in RasV12-transformed cells that are surrounded by normal epithelial cells. (**a**–**d**) Effect of paxillin-knockdown on EPLIN accumulation (**a**,**b**) or plectin accumulation (**c**,**d**). MDCK-pTR GFP-RasV12 cells or MDCK-pTR GFP-RasV12 paxillin-shRNA1 cells were mixed with normal MDCK cells on collagen gels. Cells were fixed after 16 h incubation with tetracycline and stained with anti-EPLIN (**a**) or anti-plectin (**c**) (red), and anti-paxillin (grey) antibodies and Hoechst (blue). Scale bars, 10 μM. (**b**,**d**) Quantification of (**a**,**c**). Data are mean ± SD from three independent experiments. (**b**) *P < 0.005, (**d**) *P < 0.001; n ≧ 30 cells for each experimental condition. Values are expressed as a ratio relative to paxillin-shRNA1 (-).

**Figure 3:**
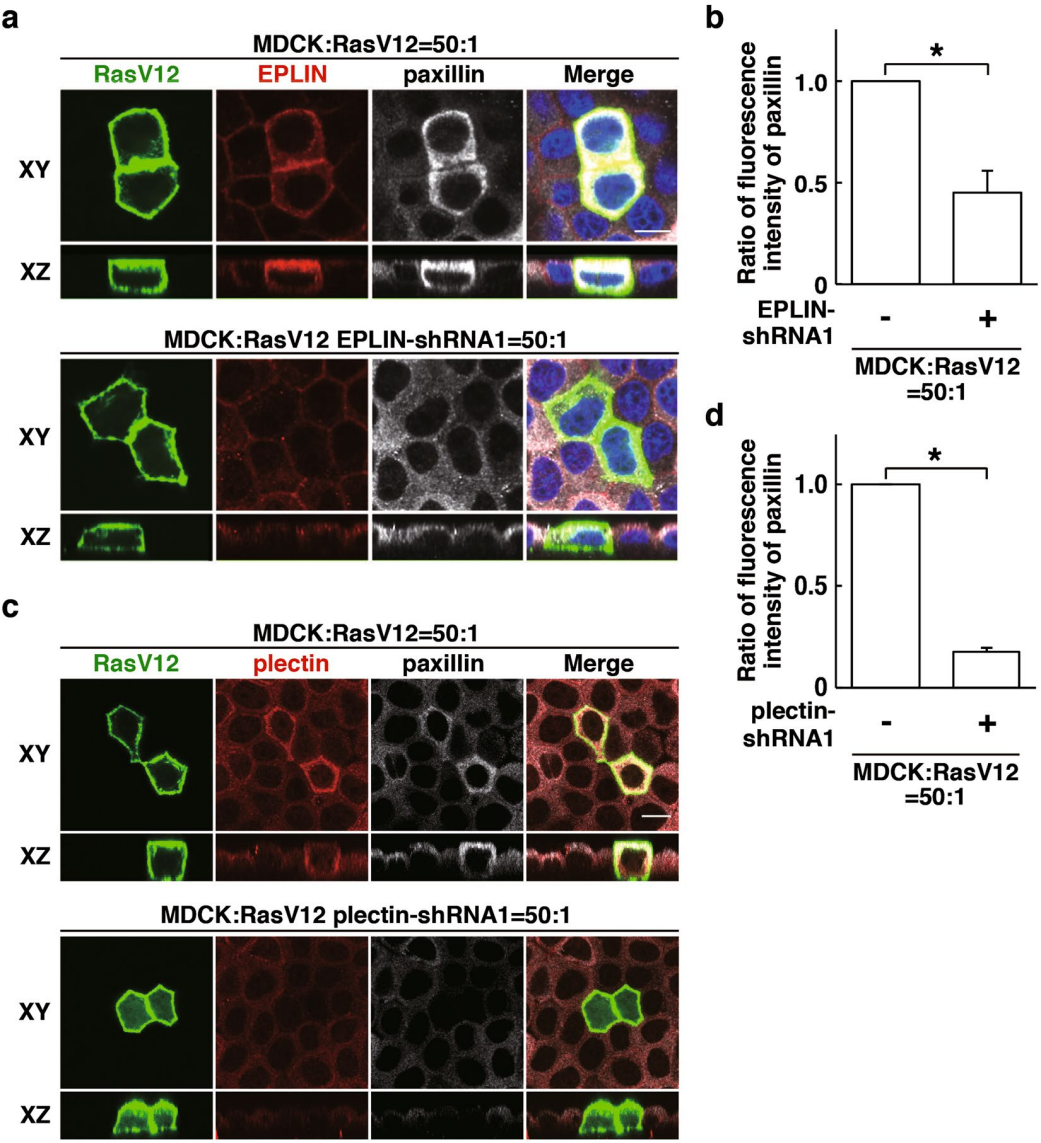
EPLIN and plectin regulate paxillin accumulation in RasV12-transformed cells surrounded by normal epithelial cells. (**a**–**d**) Effect of EPLIN-knockdown (**a**,**b**) or plectin-knockdown (**c**,**d**) on paxillin accumulation. MDCK-pTR GFP-RasV12 cells, MDCK-pTR GFP-RasV12 EPLIN-shRNA1 cells or MDCK-pTR GFP-RasV12 plectin-shRNA1 cells were mixed with normal MDCK cells on collagen gels. Cells were fixed after 16 h incubation with tetracycline and stained with anti-EPLIN (**a**) or anti-plectin (**c**) (red), and anti-paxillin (grey) antibodies and Hoechst (blue). Scale bars, 10 μM. (**b**,**d**) Quantification of (**a**,**c**). Data are mean ± SD from three independent experiments. (**b**) *P < 0.05, (**d**) *P < 0.0005; n ≧ 28 cells for each experimental condition. Values are expressed as a ratio relative to EPLIN-shRNA1 (-) (**b**) or plectin-shRNA1 (-) (**d**).

EPLIN and plectin are accumulated in RasV12-transformed cells surrounded by normal cells and play a vital role in apical extrusion of the transformed cells^14,15^. In a previous study, EPLIN was shown to interact with paxillin^27^. We thus examined the interaction between EPLIN and paxillin in our *in vitro* cell competition model system^11^. Paxillin was co-immunoprecipitated with EPLIN, and the interaction was enhanced under the mix culture condition of normal and RasV12 cells (Fig. 1a). In addition, by immunofluorescence, we demonstrated that paxillin was accumulated and partially co-localized with EPLIN in RasV12 cells that were surrounded by normal cells, but not in RasV12 cells cultured alone (Figs 1b,c, 2a and 3a).

**Figure 4:**
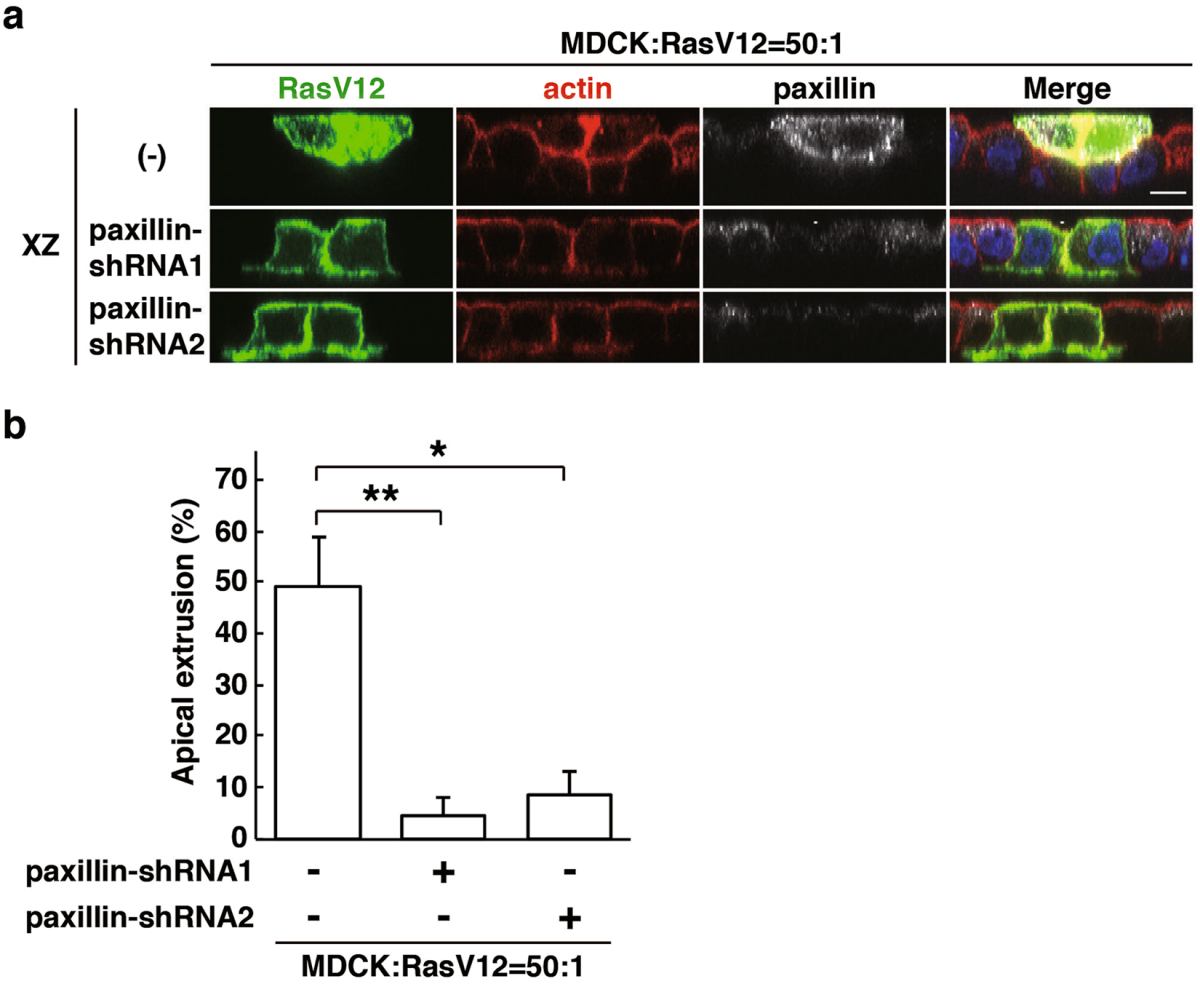
Paxillin plays a positive role in apical extrusion of RasV12-transformed cells. (**a**) XZ images of RasV12 cells or paxillin-knockdown RasV12 cells that were surrounded by normal epithelial cells. MDCK-pTR GFP-RasV12 cells, MDCK-pTR GFP-RasV12 paxillin-shRNA1 or -shRNA2 cells were mixed with normal MDCK cells. Cells were fixed after 24 h incubation with tetracycline and stained with anti-paxillin antibody (grey), Alexa-Fluor-647-phalloidin (red) and Hoechst (blue). Scale bar, 10 μM. (**b**) Quantification of the effect of paxillin-knockdown on apical extrusion of RasV12 cells. Data are mean ± SD from four independent experiments. *P < 0.005 and **P < 0.001; n = 95–135 cells for each experimental condition.

To examine the functional role of paxillin, we have established RasV12-transformed cells stably expressing paxillin-shRNA1 or -shRNA2 (Supplementary Fig. S1a). Knockdown of paxillin substantially diminished the accumulation of EPLIN in RasV12 cells that were surrounded by normal cells (Fig. 2a,b and Supplementary Fig. S1b). In addition, paxillin-knockdown also suppressed accumulation of plectin in RasV12 cells surrounded by normal cells (Fig. 2c,d and Supplementary Fig. S1c). Conversely, knockdown of EPLIN (Fig. 3a,b)^15^ or plectin (Fig. 3c,d)^14^ significantly suppressed accumulation of paxillin. When RasV12 cells were cultured alone, knockdown of paxillin or EPLIN did not affect expression of the other proteins (Supplementary Fig. S1d,e). Collectively, these results indicate that paxillin, plectin and EPLIN mutually influence their non-cell-autonomous accumulation in RasV12 cells. We next examined whether knockdown of paxillin affects the fate of RasV12-transformed cells upon cell competition with the surrounding normal cells. Knockdown of paxillin strongly suppressed apical extrusion of RasV12 cells, and most of paxillin-knockdown RasV12 cells remained within the epithelium (Fig. 4a,b), indicating that paxillin is a crucial regulator for the elimination of the transformed cells.

### Acetylation of tubulin is enhanced in RasV12-transformed cells surrounded by normal cells

**Figure 5:**
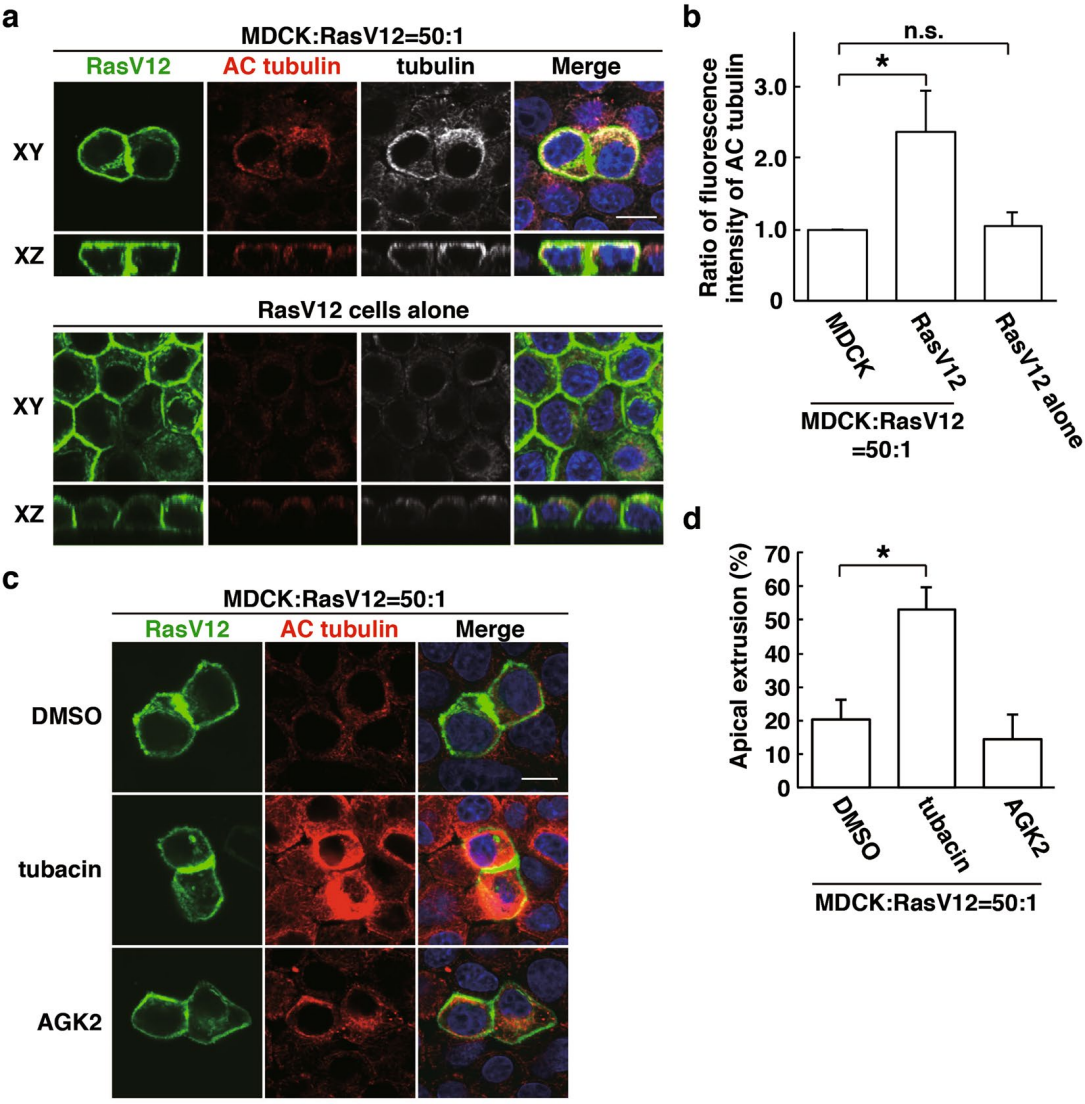
HDAC6-modulated tubulin acetylation regulates apical extrusion of RasV12-transformed cells. (**a**) Immunofluorescence images of acetylated α-tubulin. (**b**) Quantification of fluorescence intensity of acetylated α-tubulin. Fluorescence intensity of acetylated α-tubulin was analysed in each condition, and values are expressed as a ratio relative to MDCK cells in the mix culture. Data are mean ± SD from three independent experiments. *P < 0.05; n ≧ 30 cells for each experimental condition. n.s., not significant. (**c**) Effect of tubacin on tubulin acetylation in RasV12 cells. (**a**–**c**) MDCK-pTR GFP-RasV12 cells were mix-cultured with normal MDCK cells or cultured alone on collagen gels in the presence or absence of tubacin or AGK2. Cells were fixed after 16 h incubation with tetracycline and stained with anti-acetylated tubulin antibody (red) with (**a**) or without (**c**) anti-tubulin antibody (grey) and Hoechst (blue). Scale bars, 10 μM. (**d**) Quantification of the effect of tubacin or AGK2 on apical extrusion of RasV12-transformed cells. Apical extrusion was analysed after 16 h incubation with tetracycline. Data are mean ± SD from three (DMSO, tubacin) or two (AGK2) independent experiments. *P < 0.01; n = 101–176 cells for each experimental condition.

In a previous study, we have reported that α-tubulin, a major component of microtubules, accumulates at the apical domain of RasV12-transformed cells that are surrounded by normal cells^14^. Plectin and EPLIN regulate the accumulation of α-tubulin, but the molecular linkage between the plectin-EPLIN complex and tubulin accumulation remains unclear. The organization of microtubule filaments is often regulated by tubulin acetylation^16,17^. We thus examined acetylation of α-tubulin by immunofluorescence. In RasV12-transformed cells, accumulation of acetylated tubulin was mainly observed in the apical region, which overlapped with that of tubulin (Fig. 5a). Moreover, acetylation of α-tubulin was substantially elevated when RasV12 cells were surrounded by normal cells, compared with that in RasV12 cells cultured alone (Fig. 5a,b), indicating the non-cell-autonomous upregulation of tubulin acetylation in RasV12 cells. Acetylation of α-tubulin can be regulated by deacetylases: HDAC6 and SIRT2^14^. We then examined the effect of the inhibitor for HDAC6 (tubacin) or SIRT2 (AGK2). Acetylation of α-tubulin in RasV12 cells was strongly enhanced by tubacin, but not by AGK2 (Fig. 5c). Furthermore, treatment of tubacin, but not AGK2, significantly promoted apical extrusion of RasV12 cells (Fig. 5d), suggesting that acetylation of tubulin in RasV12 cells may be regulated by HDAC6, which plays a positive role in the elimination of transformed cells from epithelia.

### Paxillin regulates tubulin acetylation thereby promoting apical extrusion of RasV12-transformed cells

**Figure 6:**
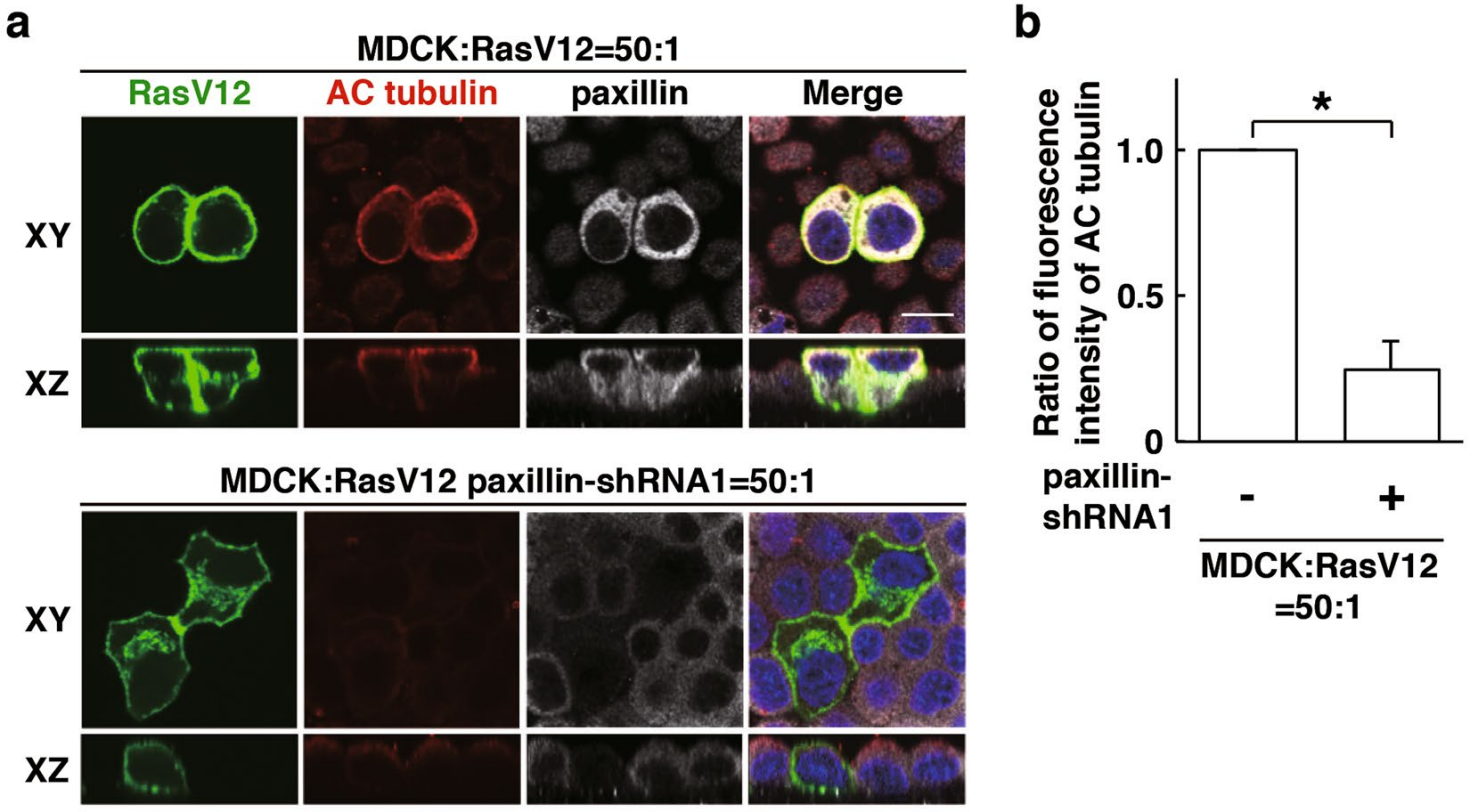
Paxillin regulates accumulation of acetylated tubulin in RasV12-transformed cells surrounded by normal cells. (**a**) Effect of paxillin-knockdown on accumulation of acetylated α-tubulin. MDCK-pTR GFP-RasV12 cells or MDCK-pTR GFP-RasV12 paxillin-shRNA1 cells were mixed with normal MDCK cells on collagen gels. Cells were fixed after 16 h incubation with tetracycline and stained with anti-acetylated α-tubulin (red) and anti-paxillin (grey) antibodies and Hoechst (blue). Scale bar, 10 μM. (**b**) Quantification of (**a**). Data are mean ± SD from three independent experiments. *P < 0.01; n ≧ 30 cells for each experimental condition. Values are expressed as a ratio relative to paxillin-shRNA1 (-).

**Figure 7:**
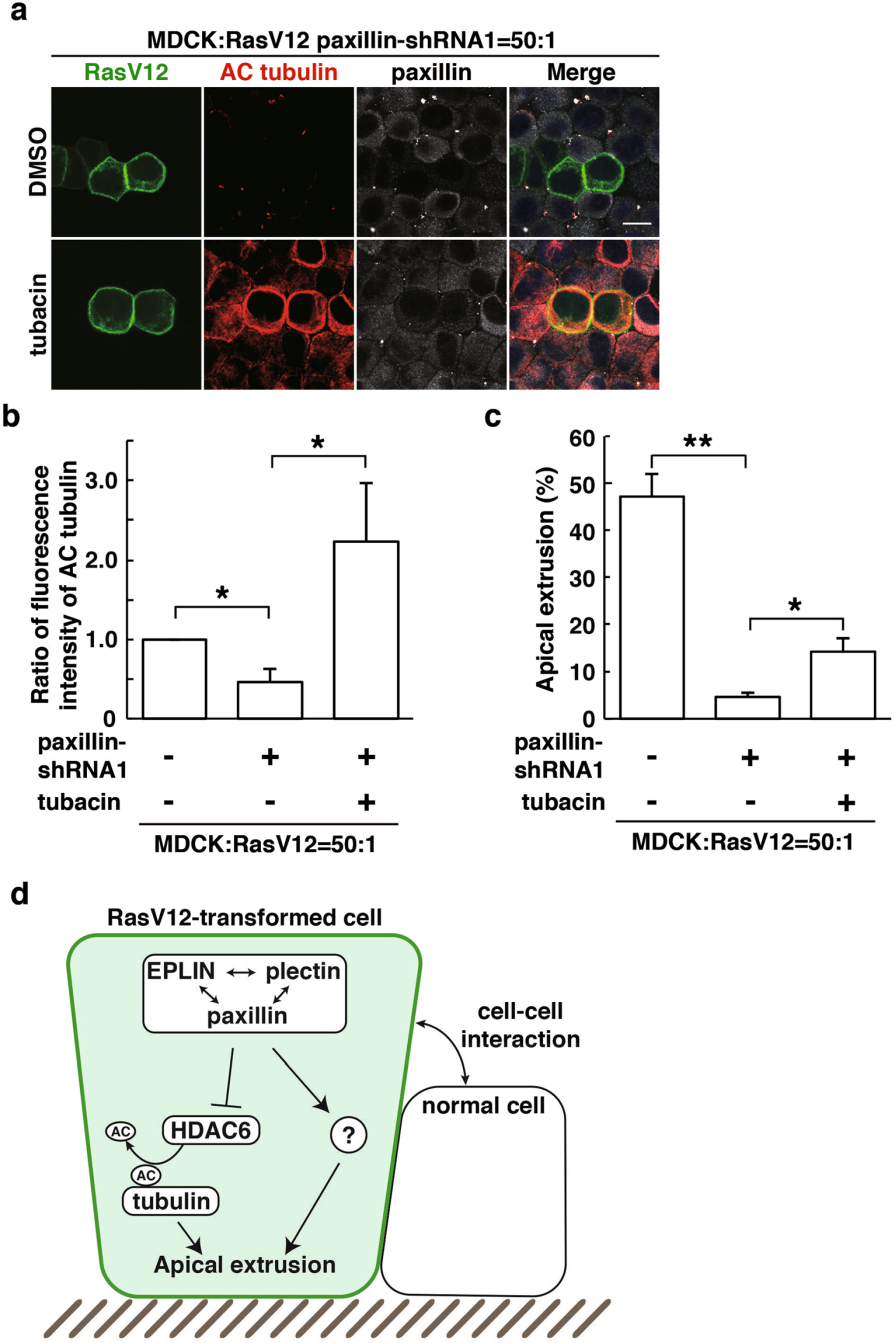
Inhibition of HDAC6 partially rescues the paxillin-knockdown phenotype. (**a**) Effect of tubacin on accumulation of acetylated α-tubulin in paxillin-knockdown RasV12-transformed cells. MDCK-pTR GFP-RasV12 paxillin-shRNA1 cells were mixed with normal MDCK cells on collagen gels. Cells were fixed after 24 h incubation with tetracycline in the presence or absence of tubacin and stained with anti-acetylated α-tubulin (red) and anti-paxillin (grey) antibodies and Hoechst (blue). Scale bar, 10 μM. (**b**) Quantification of fluorescence intensity of acetylated α-tubulin. Fluorescence intensity of acetylated α-tubulin was analysed in each condition, and values are expressed as a ratio relative to paxillin-shRNA1 (-) tubacin (-). Data are mean ± SD from three independent experiments. *P < 0.05; n ≧ 30 cells for each experimental condition. (**c**) Quantification of the effect of tubacin on apical extrusion of RasV12 paxillin-shRNA1 cells. Apical extrusion was analysed after 24 h incubation with tetracycline. Data are mean ± SD from three independent experiments. *P < 0.05 and **P < 0.005; n ≧ 100 cells for each experimental condition. (**d**) The schematic of non-cell-autonomous changes in RasV12-transformed cells neighbouring normal epithelial cells.

A previous study demonstrated that paxillin positively regulates acetylation of α-tubulin by suppressing HDAC6^25^. We found that paxillin was partially colocalised with acetylated α-tubulin in RasV12 cells that were surrounded by normal cells (Fig. 6a). In addition, knockdown of paxillin profoundly diminished the accumulation of acetylated tubulin (Fig. 6b), demonstrating that paxillin is a crucial upstream regulator of tubulin acetylation upon cell competition between normal and RasV12 cells. Knockdown of EPLIN or plectin also significantly suppressed the accumulation of acetylated tubulin (Supplementary Fig. S2a,b). Furthermore, tubacin restored accumulation of acetylated tubulin in paxillin-knockdown cells (Fig. 7a,b), suggesting that paxillin regulates tubulin acetylation by suppressing HDAC6. Moreover, tubacin partially rescued the inhibitory effect of paxillin-knockdown on the apical extrusion of RasV12 cells (Fig. 7c). Collectively, these data indicate that HDAC6-regulated tubulin acetylation is one of the downstream effectors of the paxillin-plectin-EPLIN complex in the apical elimination of transformed cells (Fig. 7d).

## Discussion

In this study, we have demonstrated that paxillin is a novel regulator for the elimination of RasV12-transformed cells from the epithelium. Paxillin regulates the accumulation of other regulators plectin and EPLIN, and *vice versa*. In addition, paxillin, in concert with plectin and EPLIN, induces acetylation of α-tubulin, leading to reorganization of microtubule filaments, at least partly, via HDAC6 (Fig. 7d). Plectin and paxillin bind to microtubules and/or HDAC6; thus the paxillin-plectin-EPLIN complex could act as a scaffolding-platform that efficiently induces the HDAC6-mediated acetylation of tubulin. However, suppression of the HDAC6 activity only partially rescued the inhibitory effect of paxillin-knockdown on the apical extrusion of RasV12 cells, suggesting that other molecules may also function downstream of the paxillin-plectin-EPLIN complex.

It still remains unclear how the accumulation of the paxillin-plectin-EPLIN complex is regulated and how tubulin acetylation positively regulates apical extrusion of transformed cells. We have previously reported that at the boundary between normal and transformed epithelial cells, various non-cell autonomous changes occur in both cells. For example, Rab5-mediated endocytosis is enhanced in RasV12 cells when they are surrounded by normal cells^28^. In addition, Warburg effect-like metabolic changes, increased glycolysis and decreased mitochondrial activity, occur in RasV12 cells neighbouring normal cells^29^. Furthermore, cytoskeletal proteins filamin and vimentin accumulate in normal cells at the interface with transformed cells, providing physical forces for apical extrusion^12^. In future studies, it needs to be elucidated whether and how these processes function upstream or downstream of the paxillin-plectin-EPLIN complex.

Apical extrusion of transformed cells can be observed *in vivo* as well, and the extruded transformed cells disappear from the tissues^29^, implying that apical extrusion is a cancer preventive phenomenon. Therefore, the molecules governing this process could be potential therapeutic targets for cancer preventive medicine. Expression of HDAC6 is upregulated in various cancer cells^30,31^. In addition, inhibition of the HDAC6 activity can suppress tumourigenesis and diminish tumor cell migration^32,33^. Thus, HDAC6 currently attracts substantial attention as one of the potential drug targets for cancer treatment^34–36^. Our data suggest that HDAC6 inhibitor could facilitate the eradication of potentially precancerous cells at the initial stage of carcinogenesis, implying that HDAC6 inhibitor can be applied not only to cancer treatment, but also cancer prevention. Further understanding of cytoskeletal organization machineries in apical extrusion would represent an interesting challenge for biological fields and cancer preventive medicine.

## Materials and Methods

### Antibodies and Materials

Mouse anti-acetylated tubulin (T6793) antibody was purchased from Sigma-Aldrich. Rat anti-α-tubulin (YOL1/34) antibody was from Abcam. Rabbit anti-paxillin (sc-5574) and mouse anti-EPLIN (sc-136399) antibodies were from Santa Cruz Biotechnology. Mouse anti-paxillin (clone 349) antibody was from BD Transduction Laboratories. Rabbit and mouse anti-paxillin antibodies were used in Figs 1b, 2a, 3a, 6a and 7a, S1c and Figs 1a, 2c, 3c, 4a, S1a,b,d,e respectively. Mouse anti-GAPDH (Clone 6C5) antibody was from Millipore. Rabbit polyclonal affinity-purified anti-plectin antibody was generated as previously described^14^. Alexa-Fluor-568- and -647-conjugated secondary antibodies were from ThermoFisher Scientific. Hoechst 33342 (Life Technologies) was used at a dilution of 1:5,000. For immunofluorescence, the primary antibodies described above were diluted in phosphate-buffered saline (PBS) containing 1% BSA at 1:100, except anti-α-tubulin antibody at 1:200, and anti-paxillin and EPLIN antibodies at 1:50. All secondary antibodies were used at 1:200. Alexa-Fluor-647-conjugated phalloidin (Life Technologies) was used at 1.0 U ml^−1^. For western blotting, primary antibodies were used at 1:1,000, except anti-α-GAPDH antibody at 1:2,000, and secondary antibodies were used at 1:1,000.

The following inhibitors were used where indicated: tubacin (Sigma-Aldrich, 10 μM) and AGK2 (Sigma-Aldrich, 10 μM). DMSO (Sigma-Aldrich) was added as a control.

### Cell Culture

MDCK and MDCK-pTR GFP-RasV12 cells were cultured as previously described^11^. MDCK-pTR GFP-RasV12 cells stably expressing EPLIN-shRNA or plectin-shRNA were established as previously described^14,15^. MDCK-pTR GFP-RasV12 cells stably expressing paxillin-shRNA were established as follows:

Double-stranded DNA fragments coding paxillin-shRNA sequences (paxillin-shRNA1:

5′-GATCCCCGCCTACAGTCTGACCTGAATTCAAGAGATTCAGGTCAGACTGTAGGCTTTTTC-3′ and

5′-TCGAGAAAAAGCCTACAGTCTGACCTGAATCTCTTGAATTCAGGTCAGACTGTAGGCGGG-3′

or paxillin-shRNA2:

5′-GATCCCCGCTTACTGCCGGAAGGATTTTCAAGAGAAATCCTTCCGGCAGTAAGCTTTTTC-3′ and

5′-TCGAGAAAAAGCTTACTGCCGGAAGGATTTCTCTTGAAAATCCTTCCGGCAGTAAGCGGG-3′)

were inserted into the *Bgl*II and *Xho*I site of pSUPER.neo + gfp (Oligoengine). MDCK-pTR GFP-RasV12 cells were transfected with pSUPER.neo + gfp paxillin-shRNA1 or -shRNA2 using Lipofectamine 2000 (Invitrogen), followed by antibiotic selection in the medium containing 5 μg ml^−1^ blasticidin (InvivoGen), 400 μg ml^−1^ zeocin (InvivoGen), and 800 μg ml^−1^ G418 (Life Technologies).

To induce the expression of GFP-RasV12, the tetracycline-inducible MDCK-pTR GFP-RasV12 cell lines were treated with 2 μg ml^−1^ tetracycline (Sigma-Aldrich). For inhibitor treatment, the indicated inhibitors were simultaneously added together with tetracycline, and then cells were further cultured for 16 h or 24 h. For immunofluorescence, cells were seeded onto Type-I collagen-mounted coverslips as described below in the section of immunofluorescence.

### Immunofluorescence

MDCK-pTR GFP-RasV12 cells were mixed with MDCK cells at a ratio of 1:50 and cultured on the collagen matrix as previously described^11^. For immunofluorescence analyses, the mixture of cells was incubated for 8–12 h until they formed a monolayer, followed by tetracycline treatment for 16 h. Cells were fixed with 4% paraformaldehyde in PBS and permeabilized with 0.5% Triton X-100 in PBS, followed by blocking with 1% BSA in PBS. Primary or secondary antibodies were incubated for 2 h or 1 h, respectively at ambient temperature. Immunofluorescence images were acquired by the Olympus FV1200 system and the Olympus FV10-ASW software. For quantification of immunofluorescence intensity, 30 transformed cells were analysed for each experiment using the MetaMorph software (Molecular Devices). For analyses of apical extrusions, the samples were prepared as described above, except that cells were treated with tetracycline for 24 h (for Fig. 5d, apical extrusion was observed after 16 h of tetracycline). More than 95 cells were analysed for each experiment, and apically extruded cells were quantified.

### Immunoprecipitation and western blotting

For immunoprecipitation, 0.6 × 10^7^ MDCK cells and 0.6 × 10^7^ MDCK-pTR GFP-RasV12 cells for mix culture or 1.2 × 10^7^ MDCK or MDCK-pTR GFP-RasV12 cells for single culture were seeded in 14.5-cm dishes (two dishes for each experimental condition) (Greiner-Bio-One) and cultured at 37 °C for 6–8 h until a monolayer was formed. Tetracycline was then added to induce RasV12 expression. After 16 h culture with tetracycline, cells were washed with ice-cold PBS containing 1 mM Na_3_VO_4_ and lysed for 30 min in NP-40 lysis buffer (20 mM Tris-HCl [pH 7.5], 150 mM NaCl and 1% NP-40) containing the following inhibitors: 1 mM Na_3_VO_4_, 0.1 mM Na_2_MoO_4_, 10 mM NaF, 5 μg ml^−1^ leupeptin, 1 mM phenylmethylsulfonyl fluoride and 7.2 trypsin inhibitor units of aprotinin. After centrifugation of the cell lysates at 21,500 g for 10 min, the supernatant was first pre-cleared with sepharose 4B (Sigma-Aldrich) at 4 °C for 30 min. This step was repeated three times. The pre-cleared cell lysates were then incubated with control IgG-conjugated Dynabeads protein G (Life Technologies) at 4 °C for 30 min and finally subjected to immunoprecipitation for 1 h with Dynabeads Protein G conjugated to rabbit anti-paxillin antibody (10 μg). Immunoprecipitated proteins were subjected to SDS-PAGE, followed by western blotting with the indicated antibodies. Western blotting data were acquired using ImageQuant^TM^ LAS4010 (GE healthcare). To examine the efficiency of paxillin-knockdown, MDCK-pTR GFP-RasV12 cells stably expressing paxillin-shRNA were seeded onto 6-cm dishes (Greiner-Bio-One) at the density of 1 × 10^6^ cells. After 24 h, the incubated cells were lysed with Triton X-100 lysis buffer (20 mM Tris-HCl [pH 7.5], 150 mM NaCl and 1% Triton X-100) containing protease inhibitors (5 μg ml^−1^ leupeptin, 1 mM phenylmethylsulfonyl fluoride and 7.2 trypsin inhibitor units of aprotinin) and directly boiled with SDS-PAGE sample buffer.

### Data Analyses

Two-tailed Student’s *t*-tests were used to determine *P*-values for statistical analyses.

### Data Availability

The datasets generated during and/or analysed during the current study are available from the corresponding author on reasonable request.

## Electronic supplementary material

Supplementary Figures 1–3
