# Supplementary material for: The paxillin-plectin-EPLIN complex promotes apical elimination of RasV12-transformed cells by modulating HDAC6-regulated tubulin acetylation
Source: Sci Rep. 2018 Feb 1;8:2097. doi: 10.1038/s41598-018-20146-1 (PMC5794774; doi:10.1038/s41598-018-20146-1)
Supplement: Supplementary file 1 — Supplementary Figures 1–3 [file 41598_2018_20146_MOESM1_ESM.pdf]

Supplementary Information

The paxillin-plectin-EPLIN complex promotes apical elimination of RasV12-transformed cells by modulating HDAC6-regulated tubulin acetylation

Nobuhiro Kasai, Ailijiang Kadeer, Mihoko Kajita, Sayaka Saitoh, Susumu Ishikawa, Takeshi Maruyama and Yasuyuki Fujita

Supplementary Figure S1-3

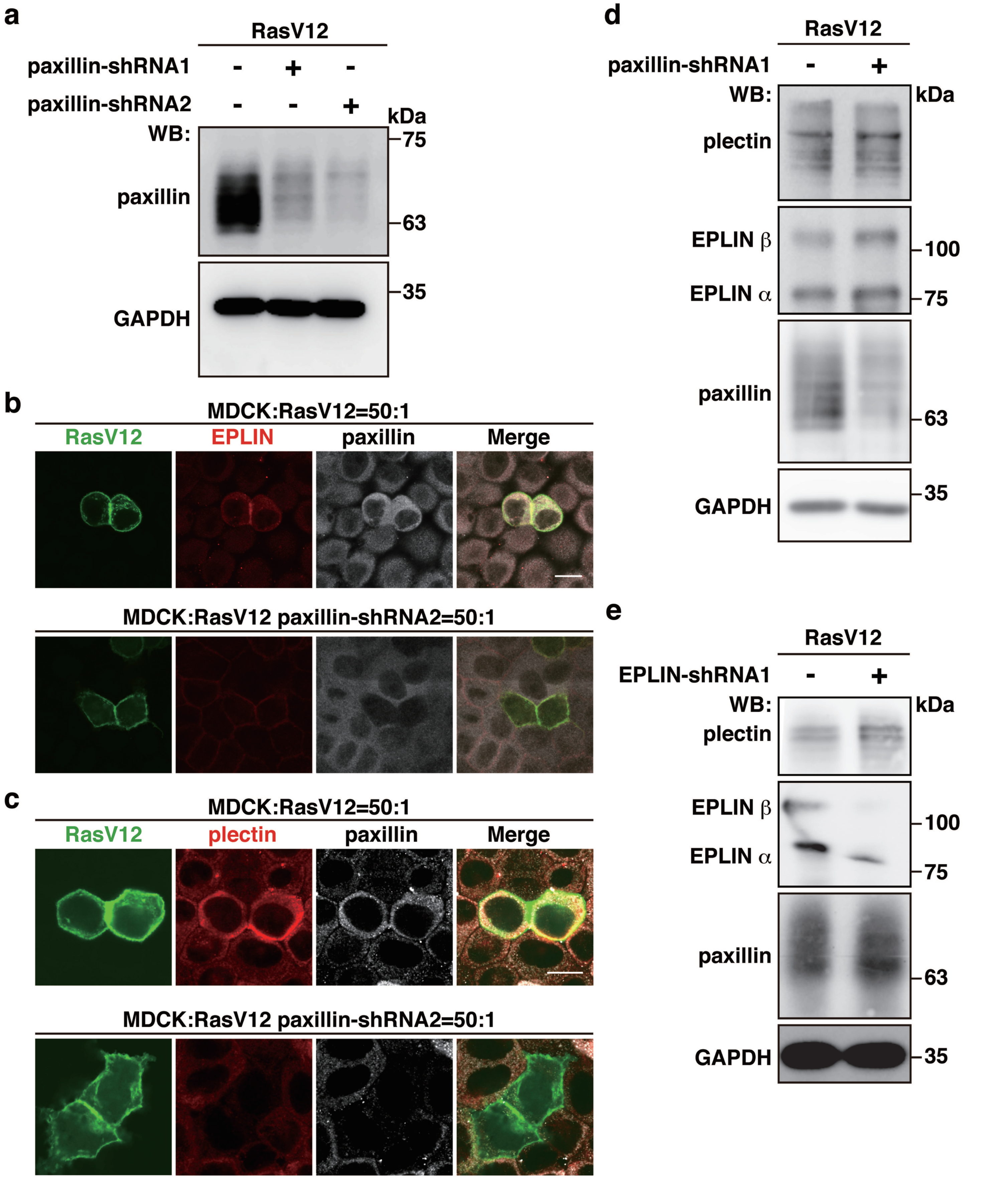

Figure S1. Paxillin regulates accumulation of EPLIN and plectin in RasV12-transformed cells that are surrounded by normal epithelial cells. (**a**) Establishment of MDCK-pTR GFP-RasV12 cells stably expressing paxillin-shRNA1 or -shRNA2. Expression of GFP-RasV12 was induced with tetracycline treatment, followed by western blotting with the indicated antibodies. (**b**,**c**) Effect of paxillin-knockdown on EPLIN accumulation (**b**) or plectin accumulation (**c**). MDCK-pTR GFP-RasV12 cells or MDCK-pTR GFP-RasV12 paxillin-shRNA2 cells were mixed with normal MDCK cells on collagen gels. Cells were fixed after 16 h incubation with tetracycline and stained with anti-EPLIN (**b**) or anti-plectin (**c**) (red), and anti-paxillin (grey) antibodies and Hoechst (blue). Scale bars, 10 µm. (**d**, **e**) Effect of paxillin- or EPLIN-knockdown on expression of paxillin, plectin and EPLIN in MDCK-pTR GFP-RasV12 cells cultured alone. Cell lysates of the parental or knockdown cells were analysed by western blotting with the indicated antibodies.

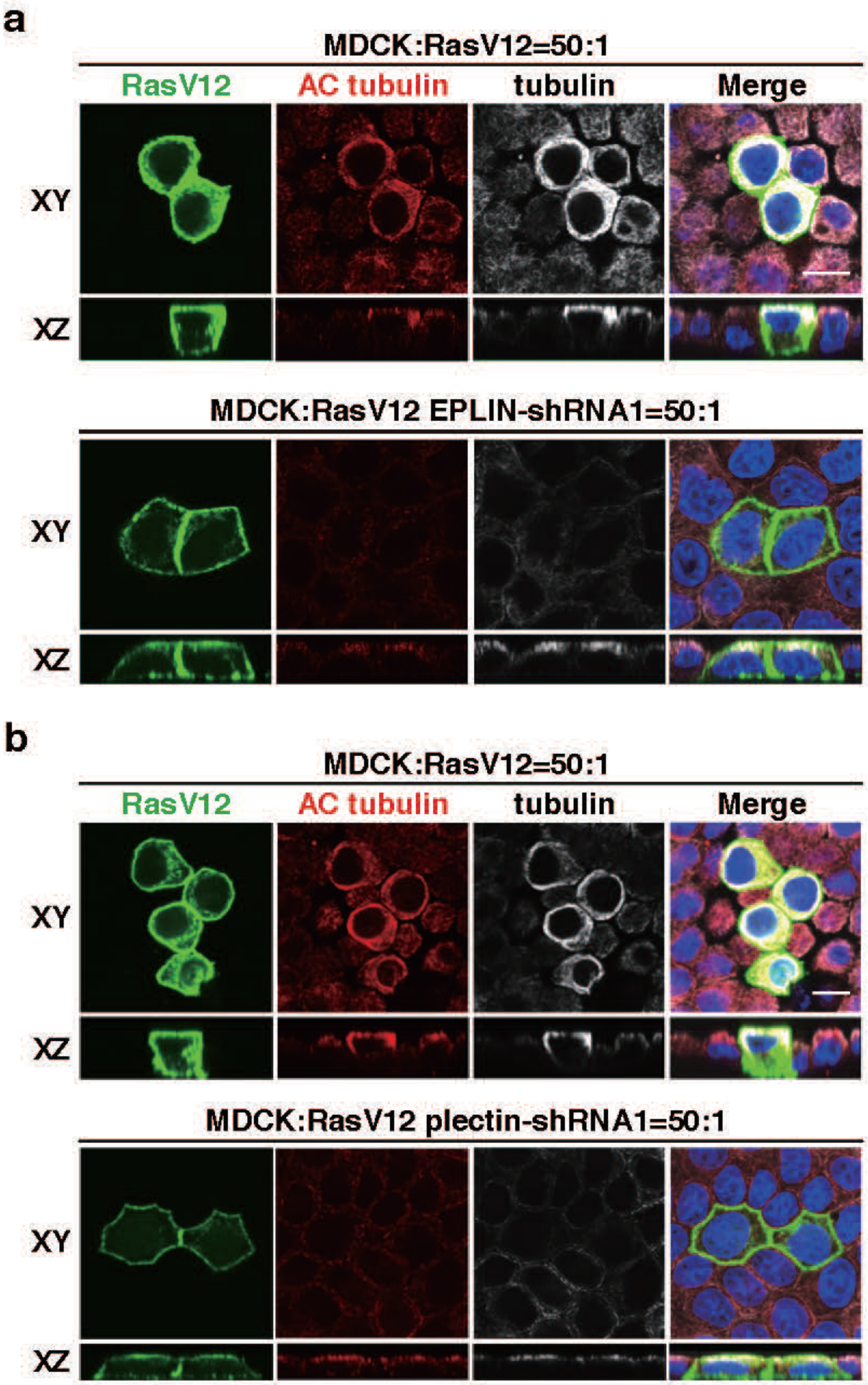

Figure S2. EPLIN and plectin regulate accumulation of acetylated α-tubulin in RasV12-transformed cells surrounded by normal epithelial cells. (**a**,**b**) Effect of EPLIN-knockdown (**a**) or plectin-knockdown (**b**) on accumulation of acetylated tubulin. MDCK-pTR GFP-RasV12 cells, MDCK-pTR GFP-RasV12 EPLIN-shRNA1 cells or MDCK-pTR GFP-RasV12 plectin-shRNA1 cells were mixed with normal MDCK cells on collagen gels. Cells were fixed after 16 h incubation with tetracycline and stained with anti-acetylated α-tubulin (red) and anti-α-tubulin (grey) antibodies and Hoechst (blue). Scale bars, 10 μm.

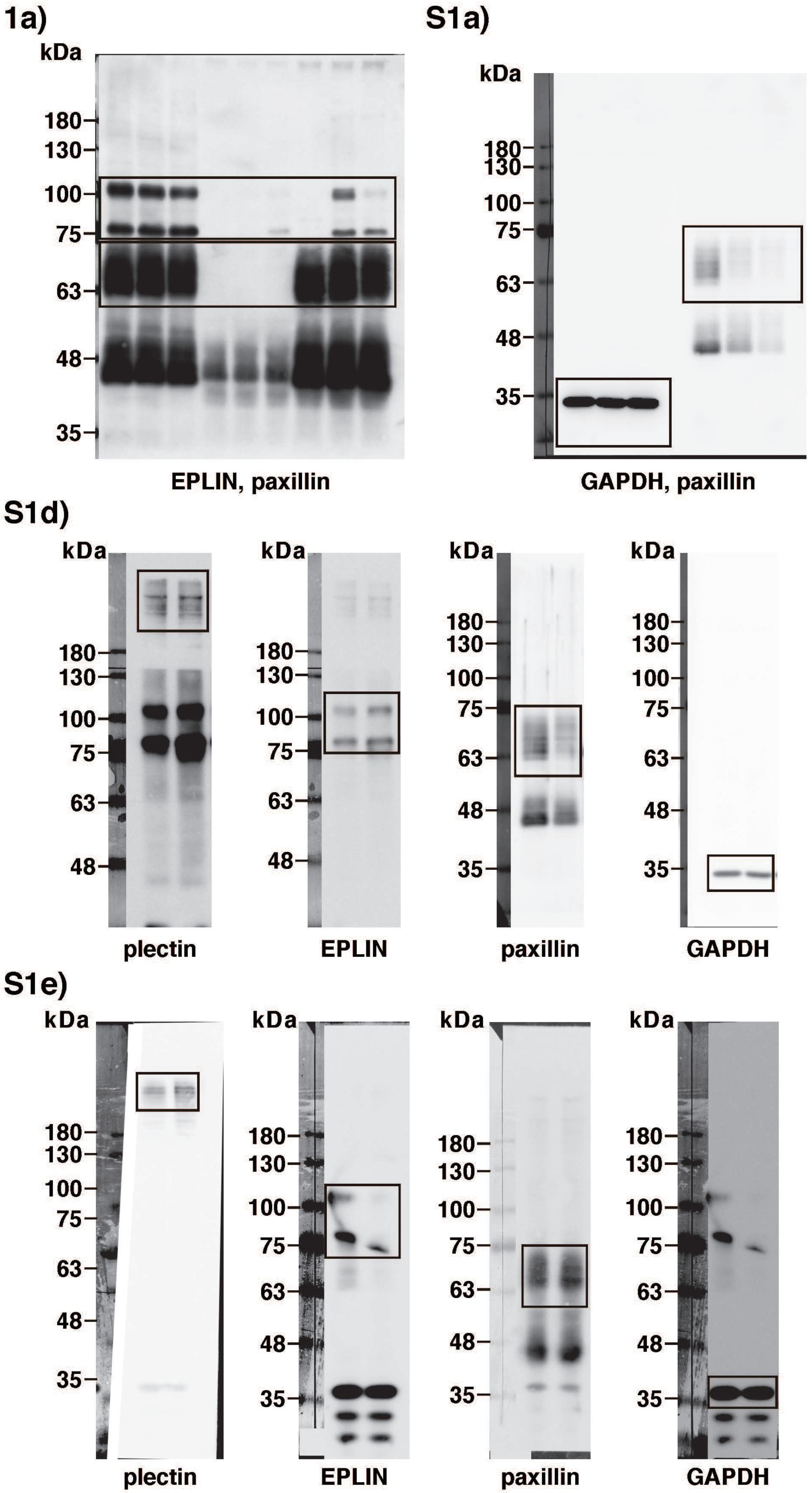

Figure S3. Full-length gels and blots.
